# Detectable Unmetabolized Folic Acid and Elevated Folate Concentrations in Folic Acid-Supplemented Canadian Children With Sickle Cell Disease

**DOI:** 10.3389/fnut.2021.642306

**Published:** 2021-04-21

**Authors:** Brock A. Williams, Cara Mayer, Heather McCartney, Angela M. Devlin, Yvonne Lamers, Suzanne M. Vercauteren, John K. Wu, Crystal D. Karakochuk

**Affiliations:** ^1^Food, Nutrition, and Health, The University of British Columbia, Vancouver, BC, Canada; ^2^BC Children's Hospital Research Institute, Vancouver, BC, Canada; ^3^Department of Pediatrics, Faculty of Medicine, The University of British Columbia, Vancouver, BC, Canada; ^4^Division of Hematopathology, Department of Pathology and Laboratory Medicine, BC Children's Hospital, Vancouver, BC, Canada

**Keywords:** sickle cell disease, pediatrics, folate, unmetabolized folic acid, B-vitamins, nutrition

## Abstract

Sickle cell disease (SCD) is an inherited hemoglobinopathy caused by a variant (rs344) in the *HBB* gene encoding the β-globin subunit of hemoglobin. Chronic hemolytic anemia and increased erythropoiesis and RBC turnover in individuals with SCD can result in increased needs for folate and other B-vitamins. We assessed B-vitamin status, and the distribution of folate forms, including unmetabolized folic acid (UMFA), in Canadian children with SCD supplemented with 1 mg/d folic acid (current routine practice). Non-fasted serum and plasma samples were analyzed for concentrations of folate, and vitamins B-2, B-6, and B-12. Eleven individuals (45% male; SCD type: HbSS *n* = 8, HbSC *n* = 2, HbSβ^0^-Thal *n* = 1), with a median (IQR) age of 14 (7, 18) years, were included. Total folate concentrations were 3–27 times above the deficiency cut-off (10 nmol/L), and 64% of children had elevated folate levels (>45.3 nmol/L). UMFA (>0.23 nmol/L) was detected in all children, and 36% of participants had elevated levels of UMFA (>5.4 nmol/L). All children were vitamin B-12 sufficient (>150 pmol/L), and the majority (55%) had sufficient B-6 status (>30 nmol/L). Among this sample of Canadian children with SCD, there was limited evidence of B-vitamin deficiencies, but UMFA was detectable in all children.

## Introduction

Sickle cell disease (SCD) is a rare, inherited hemoglobinopathy that occurs due to a missense variant (rs334) in the *HBB* gene encoding the β-globin subunit of hemoglobin (1, 2). This results in chronic hemolysis and a shortened RBC lifespan (1, 2). The increased production and turnover of RBCs in SCD is thought to increase the requirements of folate (vitamin B-9), a water-soluble family of compounds that are essential for erythropoiesis (1, 3). For this reason, individuals with SCD in Canada are routinely prescribed 1–5 mg/d of folic acid, the synthetic form of folate (4). Folic acid is commonly found in dietary supplements and folate-fortified foods, due to its stability, higher bioavailability, and oxidized state (5), while naturally occurring folate is commonly found in foods such as leafy green vegetables, oranges, beans, and legumes (6).

Beyond the role of folate in erythropoiesis, it also plays a vital role in one-carbon metabolism, a pathway which contributes to DNA and RNA synthesis, and the conversion of homocysteine to methionine in the methylation cycle (1, 6, 7). Vitamin B-2 (riboflavin), commonly found in animal products, green vegetables, and enriched grains (8), vitamin B-6, commonly found in animal products, grains, pulses, nuts and seeds (9), and vitamin B-12, found almost exclusively in animal products (10), also participate in one-carbon metabolism and are required for the production of *S*-adenosylmethionine (SAM), the universal methyl donor (11). Deficiencies of vitamins B-2, B-6, or B-12 can affect folate-dependent nucleic acid synthesis causing megaloblastic anemia, decrease cellular proliferation, and alter methylation reactions (12, 13).

A previous study of Canadian children with SCD supplemented with 1 mg/d folic acid (*n* = 92) reported that multiple serum nutrient insufficiencies/deficiencies were present in individuals, with zinc (<age- and sex-specific cut-offs) and vitamin D (<50 nmol/L) insufficiency being common (52 and 57%, respectively), vitamin B-6 deficiency (<20 nmol/L) being evident in a small minority of children (3%), but none of the children presenting with folate (<11 nmol/L) or vitamin B-12 deficiency (<133 pmol/L) (14).

Conversely, there is evidence of high folate concentrations in populations with national programs of refined-grain folic acid fortification and a high prevalence of supplement use, such as Canada and the United States. The 2011 Canadian Health Measures Survey reported that 40% of Canadians had high RBC folate concentrations (>1,360 nmol/L) (15), primarily related to supplement use (16). Unmetabolized folic acid (UMFA), folic acid that circulates in plasma when dihydrofolate reductase (DHFR) enzymatic capacity is limited or when the liver is fully saturated with folate (17), was detectable (> 0.3 nmol/L) in >95% of U.S. Americans in the 2007-8 National Health and Nutrition Examination Survey (NHANES) (18). Further, UMFA concentrations were significantly higher among supplement users (folic acid or folic acid-containing multivitamin), as compared to non-supplement users (1.54 vs. 0.79 nmol/L, respectively, *P* < 0.05) (18). Detectable UMFA concentrations in plasma have also been reported among Canadians, with 97% of pregnant women, the vast majority (≥90%) of whom consumed a prenatal supplement containing a median (IQR) of 1 (1-1) mg/d of folic acid, having detectable levels (19).

The presence of UMFA, which has been shown to occur at intake levels as low as 0.2 mg folic acid in adults (20), has not been previously investigated in children with SCD, who are routinely supplemented with high-dose folic acid (1 mg/d). This recommended dose exceeds the Tolerable Upper Intake Level (0.3–0.8 mg) for all ages between 1 and 18 years (21), and on average, the majority of Canadian children are predicted to meet the recommended dietary allowance (RDA) for folate from food sources alone in the era of folic acid food fortification (22). Further, the use of hydroxyurea, a medication which promotes fetal hemoglobin concentrations and thereby extends the average RBC lifespan in SCD (23), may reduce folate requirements. While the effects of UMFA in circulation are unknown, excess synthetic folic acid has been speculated to influence folate metabolism, DNA methylation, and gene expression (24–26).

Given the interrelationship between folate and the other B-vitamins (vitamins B-2, B-6, and B-12) in the folate and methylation cycles, and evidence of excess folic acid intake in folic acid supplemented individuals from countries with national programs of refined-grain folic acid fortification, we sought to determine B-vitamin status, and the distribution of folate forms, including UMFA, of Canadian children with SCD supplemented with prophylactic high-dose folic acid (1 mg/d).

## Materials and Methods

Non-fasted serum and plasma samples from children aged 2–19 years of age diagnosed with SCD were obtained from BC Children's Hospital BioBank (Vancouver, Canada). Blood samples were collected in vacutainer tubes with EDTA or serum separator gel, following clinical protocols. For serum, blood samples were left at room temperature for 20–30 min to allow for clotting. Blood samples, subsequently, were stored at 4°C until centrifugation (1,500 × g for 10 min at 4°C) within 3 h of sample collection. Serum and plasma aliquots were stored at −80°C until analysis.

Samples were analyzed for vitamin B-6, vitamin B-2, and folate forms using electrospray ionization-liquid chromatography-tandem mass spectrometry (Agilent Technologies; ABSciex Pte), and for vitamin B-12 concentrations using chemiluminescent immunoassay (Architect i1000, Abbott Labs.). Sufficient plasma volumes were available for vitamin B-2 and vitamin B-6 analyzes for all participants, while plasma was only available for 10 out of 11 (91%) participants for vitamin B-12 analyses and 8 out of 11 (73%) for folate analyses. In these cases, serum was used for the remaining vitamin B-12 (*n* = 1) and folate analyses (*n* = 3). Concordance correlation coefficients were calculated, based on Lin's formula (27), for individuals with both plasma and serum total folate to determine agreement between measurements. For folate form analyses, the inter-assay CVs were 10% for 5-methyltetrahydrofolate (5-MTHF), 4% for folic acid, 8% for tetrahydrofolate (THF), 4% for 5-formyltetrahydrofolate (5-FoTHF), 7% for 5,10-methlyenetetrahydrofolate (5,10-MeTHF), and 9% for 5-methyltetrahydrofolate oxidation product (MeFox). This method has been externally validated (28). For PLP and vitamin B-2 (riboflavin) analyses, the inter-assay CVs were 6.8 and 11%, respectively.

The World Health Organization (WHO) cut-offs served to determine deficiencies of folate (<10 nmol/L) and vitamin B-12 (<150 pmol/L) (29). Elevated folate concentrations were defined as >45.3 nmol/L, according to WHO cut-offs (30). Plasma UMFA >0.23 nmol/L (i.e., the lowest limit of quantification) was defined as detectable levels of UMFA, which is similar to the level of 0.3 nmol/L applied for the US population ≥1 year of age (18). Elevated UMFA was defined as >5.4 nmol/L, according to the 95th percentile of values for folic acid containing supplement users >1 year of age who had fasted venous samples collected in the NHANES 2007–2008 survey (18).

The European Food Safety Authority Panel cut-off for pyridoxal 5′-phosphate (PLP) of <30 nmol/L served to determine deficiency for vitamin B-6, as PLP is the coenzyme and main transport form of vitamin B-6 (9). As no validated cut-offs for plasma/serum riboflavin concentration to determine serum vitamin B-2 deficiency exist (8), summaries of the data are primarily presented.

Complete blood count results were collected from patient charts. Whole blood samples were analyzed for complete blood counts, including measurement of hemoglobin (Hb) concentration (g/L), hematocrit (Hct; %), mean corpuscular volume (MCV; fL), red cell distribution width (RDW; % of red blood cell), and counts of reticulocytes, platelets and neutrophils (×10^9^/L), using an automated hematology analyzer (Sysmex, Sysmex Corp.) at the BC Children's and Women's Hospital Laboratory. Megaloblastic changes were defined as a MCV increase >3 fL and a reticulocyte count <100 × 10^9^/L and/or unexplained neutropenia and thrombocytopenia. Thrombocytopenia was defined as platelets <100 × 10^9^/L (31), and neutropenia was defined as neutrophils <1.5 × 10^9^/L (32).

Patient age, sex, use of hydroxyurea, folic acid supplementation recommendations, and sickle cell genotype were gathered from patient charts. Sickle cell genotypes were classified as homozygous hemoglobin SS (β^S^β^S^), hemoglobin SC (β^S^β^C^) genotype, and hemoglobin Sβ-thalassemia genotypes.

Medians with interquartile ranges (IQR) are presented. Data were analyzed with Stata IC/16.0 for Mac (Stata Corp.). All subjects gave their informed consent for inclusion before they participated in the study. The study was conducted in accordance with the Declaration of Helsinki, and the protocol was approved by the Clinical Research Board of the University of British Columbia (#H18-01471).

## Results

In total, 11 children and adolescents with SCD (5 males; 6 females) were included in this study. Median (IQR) age of participants was 14 (7, 18) years. Overall, 8 (73%) individuals had a diagnosis of homozygous hemoglobin SS, 2 (18%) had the hemoglobin SC genotype, and 1 (9%) had the hemoglobin Sβ^0^-thalassemia genotype.

A total of 6 (55%) individuals were prescribed hydroxyurea, with a median (IQR) dose of 20 (15, 26) mg/kg/d. All individuals were recommended prophylactic folic acid supplementation (1 mg/d); adherence or duration of folic acid supplementation data was unavailable.

Megaloblastic changes were not detected in any participant (Table 1). Only 1 participant was noted to have neutropenia (neutrophils <1.5 × 10^9^) (Table 1), however, benign neutropenia has been noted to occur in individuals of African descent (32, 33).

**Table 1:** Hematological indices of 11 children with sickle cell disease^a^.

**Hematological indices**	
Hb, g/L	84 (72, 93)
Hct, %	23 (22, 26)
MCV, fL	83 (73, 91)
RDW, %	19.7 (17.2, 24.8)
Reticulocyte count, ×10^9^/L	183 (146, 255)
Platelets, ×10^9^/L	388 (240, 602)
Thrombocytopenia, platelets <100 × 10^9^/L	0 (0%)
Neutrophils, ×10^9^/L	3.9 (3.1, 6.3)
Neutropenia, neutrophils <1.5 × 10^9^/L	1 (9%)

a *Values are median (IQR) or frequency (%)*.

*Hb, hemoglobin; Hct, hematocrit; MCV, mean corpuscular volume; RDW, red blood cell distribution width*.

Concordance analysis completed for individuals with both plasma and serum total folate results (*n* = 8) illustrated a correlation coefficient (95% CI) of 0.984 (0.930, 0.996), indicating very strong agreement between serum and plasma results.

Folate deficiency (total folate <10 nmol/L) was not detected in any participant, and 7 participants had elevated folate concentrations (>45.3 nmol/L) (Table 2). Overall, folate concentrations were 3–27 times above the cut-off for deficiency. UMFA (>0.23 nmol/L; the lowest level of quantification) was detected in all 11 participants, and elevated UMFA (>5.4 nmol/L) was present in 4 participants (Table 2). UMFA as a percentage of total folate concentrations was a median (IQR) of 2.6 (2.1, 27.7) %.

**Table 2:** B-vitamin biomarkers of 11 children with sickle cell disease^a^^,^^b^.

**B-Vitamins**	
Total folate, nmol/L	62.0 (39.8, 97.6)
Folate deficient, folate <10 nmol/L	0 (0%)
Elevated folate, folate >45.3 nmol/L	7 (64%)
**Folate forms**	
MeFox, nmol/L	4.5 (3.3, 8.1)
5-MTHF, nmol/L	51.4 (29.7, 58.9)
THF, nmol/L	2.4 (1.7, 6.1)
5,10-MeTHF, nmol/L	0.3 (0.1, 0.4)
UMFA, nmol/L	1.6 (0.7, 27)
Detectable UMFA, >0.2 nmol/L	11 (100%)
Elevated UMFA, >5.4 nmol/L	4 (36%)
Vitamin B-12, pmol/L	405 (315, 478)
Vitamin B-12 <150 pmol/L	0 (0%)
PLP (Vitamin B-6), nmol/L	36.9 (29.2, 42.5)
PLP <30 nmol/L	5 (45%)
Vitamin B-2, nmol/L	15.9 (11.0, 30.5)

a *B-vitamin biomarker concentrations were measured in plasma (n = 8) and serum (n = 3) for folate forms, in plasma (n = 10) and serum (n = 1) for vitamin B-12, and in plasma for pyridoxal 5′-phosphate (n = 11) and vitamin B-2 (n = 11)*.

b *Values are median (IQR) or frequency (%). MeFox, 5-methyltetrahydrofolate oxidation product; MTHF, methyltetrahydrofolate; THF, tetrahydrofolate; MeTHF, methylenetetrahydrofolate; UMFA, unmetabolized folic acid; PLP, pyridoxal 5′-phosphate*.

**Figure 1:**
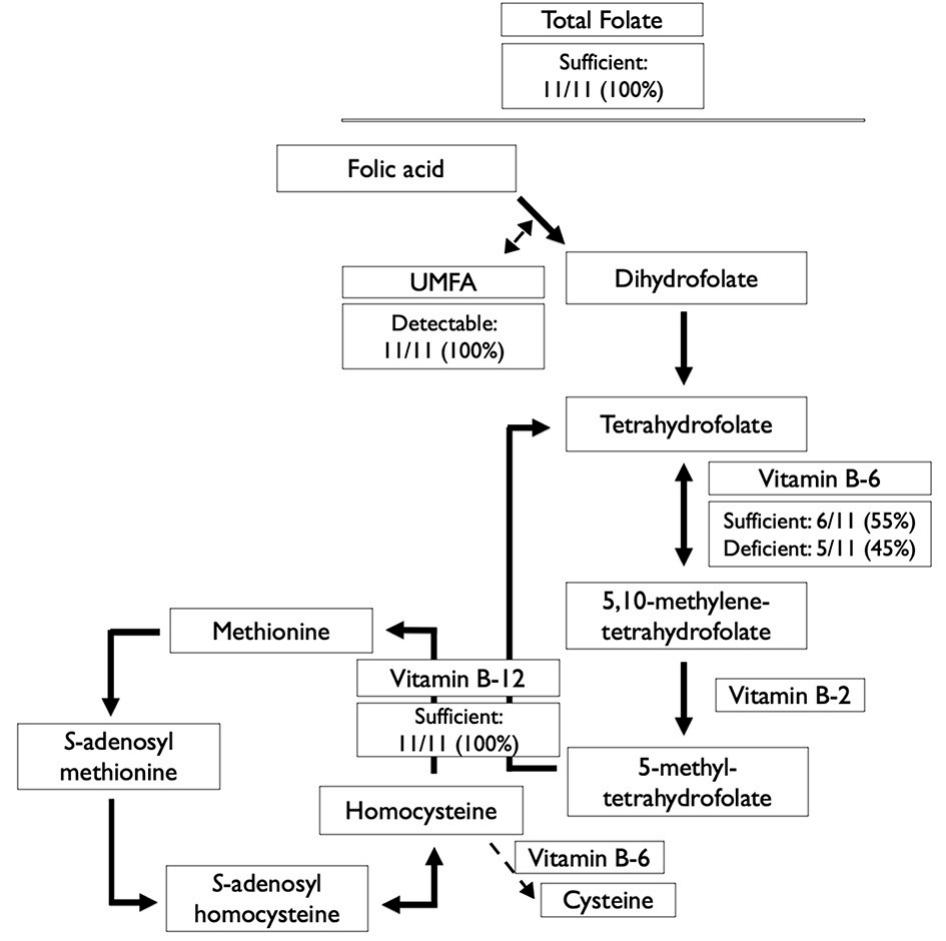
Serum/plasma status of B-vitamins involved in the folate and methionine cycles among 11 Canadian children with sickle cell disease. UMFA, unmetabolized folic acid.

There was no evidence of vitamin B-12 deficiency in this population, however, 5 individuals were vitamin B-6 deficient (PLP <30 nmol/L) (Table 2 and Figure 1).

## Discussion

Routine high-dose (1–5 mg/d) folic acid supplementation in SCD is a practice that is intended to prevent the occurrence of folate deficiency and the potential for megaloblastic anemia in this hemolytic condition. In children with SCD, this practice is largely based on a limited body of evidence consisting of one randomized control trial conducted in Jamaican children in 1983, which provided evidence of higher serum folate concentrations but no changes in hematological measures when children were supplemented with 5 mg/d of folic acid (1). The results of our study suggest that folate deficiency and megaloblastic changes were not present in this sample of Canadian children with SCD supplemented with folic acid, and that 64% of individuals had elevated folate concentrations. Further, detectable levels of UMFA were present in all participants, and there was evidence of elevated levels of UMFA in 36% of participants.

Previous studies have identified detectable UMFA and elevated folate concentrations in adult populations with hemolytic blood disorders, specifically hereditary spherocytosis (34) and β-thalassemia (35), that consumed folic acid-fortified refined grains and were supplemented with 5 mg/d of folic acid. This is the first study, to our knowledge, to report this observation in folic acid-supplemented children with sickle cell disease, which is of note as UMFA has been proposed to be a biomarker of excess folic acid intake (36). A recent National Institutes of Health (NIH) Workshop (2020) on understanding the metabolic and clinical effects of excess folates/folic acid has identified that the evidence on possible adverse effects of excess folate and/or UMFA remains inconclusive, but there is an emerging need to address these gaps in the literature (37). Previous studies have reported an association between excess folic acid intakes and colorectal (38) and prostate (39) cancer in adults, impaired cellular immunity [reduced natural killer cell cytotoxicity in adults, and potential effects on the mucosal immune tissue of the gut in children (40, 41)], and adverse effects on metabolic health during development (42, 43). This may be of significant importance for children in SCD who may be exposed to UMFA, which can occur at doses of folic acid as low as 0.2 mg/d (20), chronically from life-long folic acid supplementation of ≥1 mg/d.

As folate does not work in isolation in one-carbon metabolism, we also determined the status of other key B-vitamins (vitamins B-6 and B-12) in this supplemented population. In this sample, there was no evidence of vitamin B-12 deficiency; however, vitamin B-6 deficiency was evident in 45% of participants (*n* = 5). Previous cross-sectional studies have suggested that among US children with SCD, not treated with hydroxyurea, that 77% (*n* = 88/109) of individuals were vitamin B-6 deficient (44), while in Canadian children with SCD, the majority of whom were treated with hydroxyurea, that 19% (*n* = 15/80) were deficient (14). Of note, in both studies, a PLP concentration of <20 nmol/L was used to determine deficiency of vitamin B-6, and no participants in this study had PLP concentrations below that cut-off. Thus, the 45% of children with PLP concentration between 20 and 30 nmol/L classified as insufficient in our study, would be classified as having marginal B-6 status using previously applied adult categories (20–30 nmol/L) (45).

We present a descriptive summary of plasma vitamin B-2 for this sample of children, as no well-established cut-offs exist for determining deficiency of vitamin B-2 using plasma or serum concentrations (8). Vitamin B-2 status is most often determined using the erythrocyte glutathione reductase activation coefficient (EGRac), a functional measure of B-2 status (46), which is only available in a select few research institutions and requires washed red blood cells as biospecimen, which was not available for this sample of children with SCD. Emerging research has sought to establish the usefulness of plasma vitamin B-2 as a more-widely available indicator of B-2 status. Preliminary data among healthy women has identified a plasma concentration of 26.5 nmol/L of vitamin B-2 as the change point between plasma riboflavin and an EGRac of 1.25, indicative of functional B-2 deficiency, and a range of 6.7–64.2 nmol/L as the lower and upper limits of the central 95% reference interval, respectively (47). Among participants in our study, 73% (*n* = 8) had plasma riboflavin concentration below the change point of 26.5 nmol/L, indicative of functional B-2 deficiency. Further study which establishes the utility of plasma vitamin B-2 for the determination of B-2 status may help to provide a holistic understanding of B-vitamin status in children with hemolytic hemoglobinopathies. This is of value as adequate vitamin B-2 is important for folate cycle activity, specifically the formation of 5-MTHF, as well as for the conversion of dietary vitamin B-6 to its coenzyme form PLP (8).

While SCD is the most common monogenic disease worldwide (48), in the Canadian population it is a relatively rare disease estimated to occur in up to ~1 in 5,600 births in some regions of the country (49). We acknowledge the inherent limitation of our small sample size given the rarity of this disease, especially in Western Canada; however, in British Columbia, our sample of children with SCD represents ~20% of the total pediatric SCD population. We acknowledge a few other limitations of our study: a lack of dietary data and biomarkers such as RBC folate concentrations for the determination of longer-term folate status and EGRac for the determination of B-2 status. In general, RBC folate concentrations are considered to be reflective of longer term folate status, previous 3–4 months in healthy RBCs, whereas serum/plasma folate reflects recent status or dietary/supplementary intake (50). It has, however, been suggested that steady state RBC folate concentrations can appear higher in those with HbSS and HbSC genotypes, in comparison to healthy controls (HbAA; normal hemoglobin) supplemented with the same dose of folic acid (5 mg/d) (51). This results from the shorter RBC half-life in SCD, as RBC folate concentrations are established during erythropoiesis and subsequentially decrease in circulation (51). In this population with a higher proportion of young RBCs, a comprehensive approach to assessment of folate status, which includes both serum/plasma and RBC folate concentrations, may aid in this interpretation.

In this study, the high plasma/serum folate concentrations in non-fasting samples measured in some participants may have resulted from recent folic acid supplementation consumption. The vast majority of participants (*n* = 8) had a percentage of total folate concentrations resulting from UMFA <10%, while three participants had concentrations above that threshold (27.7, 56.8, and 71.7%, respectively), likely indicating recent ingestion of a significant amount of folic acid.

The analysis of vitamin B-12 using chemiluminescent immunoassay in SCD, a hemolytic hemoglobinopathy, may have also potentially underestimated total B-12 concentrations. One sample in our study had significant hemolysis and there is evidence to suggest that greater degrees of hemolysis can lead to lower measured vitamin B-12 in plasma when using the Abbott Architect instrument (52). The inclusion of other biomarkers, such as holotranscobalamin, the form of vitamin B-12 in circulation that is taken up by tissues, and/or methylmalonic acid, which is elevated in vitamin B-12 deficiency, may have provided a more accurate estimation of vitamin B-12 status than just plasma total vitamin B-12 alone (53).

This cross-sectional study presents preliminary evidence of detectable unmetabolized folic acid and limited B-vitamin deficiencies among this sample of Canadian children with SCD supplemented with prophylactic high-dose folic acid. Future rigorously designed, and comprehensive research in which RBC and serum folate concentrations, folate-related metabolites, such as *S*-adenosylmethionine, *S*-adenosylhomocysteine, and total homocysteine concentrations, other biomarkers for B-vitamins involved in folate metabolism, including vitamins B-6 and B-12, and biomarkers of inflammation and oxidation are measured during periods with and without high-dose folic acid supplementation for pre-defined study time periods in children with SCD may support the findings presented, allow for detailed analysis of one-carbon metabolites in these individuals, and provide further insight into the utility of routine folic acid supplementation. Larger, sufficiently powered trials are also warranted to determine if B-vitamin status differs by sickle cell genotype, the degree of RBC hemolysis, and/or the use of hydroxyurea.

## Data Availability Statement

The raw data supporting the conclusions of this article will be made available by the authors, without undue reservation.

## Ethics Statement

The studies involving human participants were reviewed and approved by Clinical Research Board of the University of British Columbia. Written informed consent to participate in this study was provided by the participants' legal guardian/next of kin.

## Author Contributions

CK, BW, HM, SV, and JW: conceptualization. CM and HM: assistance with study preparation. YL: sample analysis. BW and CK: writing—original draft preparation. CM, HM, AD, YL, SV, and JW: writing—review and editing. CK: supervision and funding acquisition. All authors have read and agreed to the published version of the manuscript.

## Conflict of Interest

The authors declare that the research was conducted in the absence of any commercial or financial relationships that could be construed as a potential conflict of interest.
